# Evaluation of the histologic and immunohistochemical (CD34, glutamine synthetase) findings in idiopathic non-cirrhotic portal hypertension (INCPH)

**DOI:** 10.1007/s12072-024-10654-w

**Published:** 2024-03-27

**Authors:** Melek Büyük, Neslihan Berker, Doğu Vurallı Bakkaloğlu, İbrahim Volkan Şenkal, Zerrin Önal, Mine Güllüoğlu

**Affiliations:** 1 Department of Pathology, Istanbul Faculty of Medicine, Istanbul University, Fatih, Istanbul, Turkey; 2 Department of Gastroenterology and Hepatology, Istanbul Faculty of Medicine, Istanbul University, Istanbul, Turkey; 3 Department of Pediatric Gastroenterology, Hepatology and Nutrition Department, Istanbul Faculty of Medicine, Istanbul University, Istanbul, Turkey

**Keywords:** Idiopathic non-cirrhotic portal hypertension, CD34, Glutamine synthetase, Histology, Liver biopsy

## Abstract

**Aim:**

Idiopathic non-cirrhotic portal hypertension (INCPH) is a vascular disorder of uncertain origin. Diagnosis can be challenging on liver biopsy. Despite diverse histomorphologic findings documented in literature, studies on the frequency of these findings are lacking. This study aims to assess both the histomorphologic features and the immunoexpression patterns of CD34 and glutamine synthetase (GS) in liver biopsies and searched for their contribution to the pathologic diagnosis of INCPH.

**Materials and methods:**

Hematoxylin–eosin, CD34, and GS-stained liver needle biopsy sections of 16 patients clinically diagnosed with INCPH were retrospectively analyzed. Histologic findings such as portal vein narrowing, obliteration, or loss were grouped as major findings, while portal vein herniation, hypervascularized portal tracts, and periportal abnormal vessels were grouped as minor findings, and their frequency were evaluated. Periportal endothelial CD34 stained areas were measured via ocular micrometer. The distribution of GS immunoexpression was evaluated. Eighteen healthy liver donor biopsies were evaluated as controls.

**Results:**

In INCPH cases, 58% of portal tracts showed major findings, compared to 15% in the control group (p < 0.001). Minor findings were observed in 16% of INCPH cases and 7% of controls (p = 0.014). The number of portal tracts with histologic findings is significantly higher in INCPH than in control liver biopsies. Abnormal portal tract distribution, like being close to each other, was seen in 75% of INCPH cases but not in controls (p < 0.001). Nodular regenerative hyperplasia (NRH) was present in 31% of cases. Periportal CD34 expression was higher in INCPH, and affected areas were larger than in controls (p < 0.001). Irregular GS staining, i.e. GS staining with patchy distribution in zone 3, and/or periportal and zone 2 hepatocytes, was found in 62% of INCPH cases, while controls showed the usual pattern (p < 0.001).

**Conclusion:**

In the biopsy diagnosis of INCPH, in addition to the presence of major histologic findings and the amount of portal tracts displaying these features, the expression of endothelial CD34 in periportal areas, and irregular hepatocellular GS expression can also be considered as supporting feature.

## Introduction

Portal hypertension (PH) has various etiologies other than cirrhosis including prehepatic, hepatic, and posthepatic conditions [1, 2]. When signs and symptoms of PH develop in the absence of liver cirrhosis, the condition is designated as non-cirrhotic portal hypertension (NCPH) [3, 4]. Many disorders are associated with NCPH, such as infiltrative diseases (i.e., sarcoidosis), vascular malignancies, schistosomiasis, congenital hepatic fibrosis, and primary liver diseases (i.e., primary biliary cholangitis, autoimmune hepatitis). The diagnosis of idiopathic non-cirrhotic portal hypertension (INCPH) is possible if all these disorders have been excluded [5, 6].

The term of INCPH was used for the first time in 2011 for the presence of PH without underlying liver disease and cirrhosis [7]. Various terms were used to date such as hepatoportal sclerosis, non-cirrhotic portal hypertension, idiopathic non-cirrhotic intrahepatic portal hypertension, idiopathic portal hypertension, non-cirrhotic portal fibrosis, incomplete septal cirrhosis, obliterative portal venopathy, and nodular regenerative hyperplasia for this entity [4, 6, 8–15]. Since similar histomorphological findings and clinical courses were observed and varying terms were confusing, the term of INCPH was introduced to standardize the nomenclature as a distinct single entity with various pathological aspects, rather than different clinicopathological entities [7].

INCPH is a vascular condition with unknown etiology causing PH and related clinical symptoms and laboratory findings [7, 16, 17]. Although the etiology is unknown, various circumstances such as immune function disorders, infectious etiologies, hematologic factors, metabolic factors, genetic predispositions, and drug exposure are thought to be playing role in disease development [3, 16, 18]. Cases without a defined underlying cause leading to INCPH were reported in 53% of a series comprising fifty-nine patients [8].

Liver biopsy is required for the diagnosis of INCPH due to the lack of definitive diagnostic tests and to rule out cirrhosis [5, 19]. Various histological changes, especially in portal/periportal areas, have been described in liver biopsies. However, INCPH may be underdiagnosed, because of inconspicuous and heterogeneously distributed histologic findings, in addition to the unawareness of the pathologists to this entity and evaluation of the biopsies without enough clinical information [6, 9–11, 18, 19]. The biopsy may appear nearly normal at low magnification. However, being aware of this diagnostic possibility and through examination of the liver biopsy may reveal some histological findings for the correct diagnosis [16–20]. Even though both portal tracts and lobular areas are affected, the most common striking features of INCPH are observed within the portal tracts. The histomorphologic findings of INCPH are summarized below:

### Portal/periportal findings

Portal tracts may be fibrotic, and the borders are rounded. Portal vein (PV) might be narrowed, obliterated, or lost. Obliterative portal venopathy (OPV), also known as phlebosclerosis, is considered to be the hallmark of INCPH [4]. OPV is characterized by mural fibrosis and thickening of intrahepatic small and medium-sized portal vein branches with subsequent luminal narrowing, obliteration and venopenia [12, 20]. Sometimes portal tract sclerosis or fibrosis cannot be seen, some portal tracts may be hypoplastic and only the remnants of the portal tracts might be present. PV branch is inconspicuous or absent in these portal tract remnants [16]. Identification of a narrowed PV in smallest or hypoplastic portal tracts may be difficult or they are overlooked [19]. Portal vein herniation into the periportal parenchyma, abnormal thin-walled periportal vessels, and portal tract hypervascularization creating a slit-like appearance are the other vascular findings of INCPH [9, 16–20].

### Parenchymal architectural changes

The most common parenchymal lesion in INCPH is sinusoidal dilatation which is not specific to INCPH. It is usually due to increased sinusoidal pressure caused by changes in arterial or portal blood flow [17]. Another parenchymal finding is the close location of portal tracts to each other. Obliteration of the portal vein leads to parenchymal hypoperfusion, resulting in parenchymal extinction defined as a region with loss of contiguous hepatocytes that cause the proximity of portal tracts to each other [9, 18, 21].

Nodular regenerative hyperplasia (NRH) is another histologic finding consisting of numerous ill-defined nodules without fibrosis throughout the liver [10, 11, 13, 18, 22]. NRH demonstrates a vaguely nodular cut surface with the nodules having a pale appearance and the borders being darker in color. The pale-appearing nodules represent the areas of hypertrophy, and the borders consist of atrophic hepatocyte plates with or without any associated congestion [18].

In normal liver, sinusoidal endothelial cells do not exhibit CD34 expression. In some conditions such as focal nodular hyperplasia, macroregenerative nodule, CD34 expression can be observed in sinusoidal endothelial cells [23]. There is limited knowledge about the endothelial CD34 expression pattern in INCPH cases. Periportal sinusoidal CD34 immunoexpression has been mentioned in a few studies in patients with NRH and OPV [24, 25].

Glutamine synthetase (GS) is a useful marker in the differential diagnosis of liver tumors including hepatocellular adenomas and nodules in cirrhosis [26, 27]**.** In addition, abnormal hepatocellular GS expression patterns have been well documented in focal nodular hyperplasia (FNH) and nodular regenerative hyperplasia (NRH) [28, 29]. Normal GS expression is defined as a rim of 2–3 layers of strongly positive hepatocytes around the hepatic veins (perivenular pattern) [30]. In NRH, besides pericentral hepatocytes, immunoreaction in zone 2 hepatocytes [29] and diffuse hepatocellular immunoreaction were also reported [31].

Because of the ambiguous and heterogeneously distributed histologic findings and the lack of the entire set of histopathologic findings in the same biopsy, diagnosis of INCPH may be difficult, especially in small biopsies considered as nearly adequate. Portal venous obliteration, portal vein loss, and portal tract sclerosis were described as common findings. However, there are no well-established diagnostic criteria considered adequate for the histopathologic diagnosis and the amount of portal tracts displaying these findings have not been examined in detail in the literature. Furthermore, there is limited information about the utility of immunohistochemistry on the diagnosis of INCPH.

In this study, our aim was to evaluate all the histologic features as well as the endothelial CD34 and hepatocellular GS expression patterns in detail and reveal their impact on the biopsy diagnosis of INCPH.

## Materials and methods

### Patients

Liver biopsies of the patients with the clinical diagnosis of INCPH between the years 2015–2022 were included in this study. After the patients without clinical data were excluded, a total of 16 patients with NCPH of unknown etiology were studied.

Eighteen living-related liver transplant donor biopsies were included as control group. The liver biopsies of both study and control cases were obtained percutaneously under ultrasound guidance.

### Histologic evaluation

All archived Hematoxylin–Eosin, Masson trichrome (MT), and reticulin stained liver biopsy slides were re-examined, and histologic findings were documented. Portal tracts were counted. Portal/periportal vascular changes were examined. Because OPV findings have been accepted as the hallmark of INCPH [4], portal vein narrowing, obliteration, and/or loss (complete or incomplete) were grouped as major findings, while portal vein herniation, hypervascularized portal tracts, and/or periportal abnormal vessels were grouped as minor findings, and the percentages of histologic findings were documented for each biopsy. The presence of any of the major or minor histologic finding was considered. The findings such as sinusoidal dilatation, the proximity of portal tracts to each other, and nodular regenerative hyperplasia were also evaluated. Two experienced liver pathologists (MG, MB) evaluated the biopsy sections by reaching a consensus on histologic findings.

### Immunohistochemistry

Four-µm thick tissue sections of INCPH and control cases were incubated with the primary antibodies CD34 (qbend/10, Novocastra, 1 h incubation) and Glutamine synthetase (6/GS, Biocare, 1 h incubation), by using an automated staining module (Ventana Medical System-Benchmark XT/ISH Staining Module, Roche, Switzerland). Results were evaluated by two experienced liver pathologists (MG, MB).

### Immunohistochemical evaluation

CD34 immunoreaction was detected in hepatic and portal venules as well as periportal and periseptal sinusoids in some cases. Presence of sinusoidal endothelial immunoreaction was considered sinusoidal capillarization and the extension of the stained area (from the portal tract edges to the parenchyma) was measured via an ocular micrometer under light microscope.

Normal GS expression was defined as a rim of 2–3 layers of strongly positive hepatocytes around the hepatic veins (perivenular pattern) [30]. GS staining with patchy distribution in zone 3, and/or periportal and zone 2 hepatocytes was considered as irregular staining. Multizonal staining (staining in both zone 2 and zone 3) was considered as diffuse irregular staining.

### Statistical analysis

All statistical analyses were performed using the Statistical Package for Social Sciences (SPSS) software for Windows version 22.0 (IBM Corp, Armonk, NY, USA). For normalization of data Kolmogorov–Smirnov test was applied. Descriptive statistics were displayed as mean ± SD and categorical variables as frequency and percentage values (%). The importance of the difference between the case and control groups in terms of mean values was evaluated with independent sample T test. Categorical variables were evaluated using Pearson’s chi-square test. p value of less than 0.05 was considered statistically significant.

## Results

### Patient characteristics

The study cohort included 6 males (37.5%) and 10 females (62.5%). The mean age was 34 (± 2 SD; range 4–60) years. All cases included in the study cohort had a clinical diagnosis of portal hypertension. Six cases had only esophageal varices at upper endoscopy, and nine cases had esophageal varices and splenomegaly, and one case had bleeding esophageal varices and ascites. The portal vein thrombosis was ruled out by Doppler-ultrasound in all patients. None of the patients had definite clinical history or histologic evidence of underlying liver diseases such as viral hepatitis, autoimmune hepatitis, fatty liver disease, cholestatic liver disease.

In the control group, the mean age was 38 (± 11 SD; range 23–62) years and included 8 males (44.5%) and 10 females (55.5%).

Clinical characteristics of INCPH and control cases are shown in Table 1**.**

**Table 1:** Clinical and histopathological characteristics of INCPH and control cases

	INCPH (*n*: 16)	Control cases (*n*: 18)
Age, year (mean)	34 (4–60)	38 (23–62)
Female/male	10/6	10/8
Biopsy indication		
PH, splenomegaly	9	Healthy living liver donors
PH, elevation of liver enzymes	5	
PH, suspicious of chronic liver disease	1	
PH, suspicious of cirrhosis	1	
Histopathologic diagnosis		
Portal venopathy	11	Normal histologic findings
Suggestive of portal venopathy	2	
Mild sinusoidal dilation	1	
Normal histological findings	1	
Inadequate biopsy	1	

*PH* portal hypertension

### Histologic findings

The mean size of the liver biopsies was 1.9 cm (range 0.6–3 cm) in the study group, and 2.1 cm (range 1–3 cm) in the control group cases. The mean number of portal tracts was 18 (range 7–40) in the study group, and 16 (range 7–25) in the control group. Major findings were observed in all 16 INCPH cases (100%) while 15 (83%) of the control group cases displayed these findings. The number of portal tracts with major findings showed variation among the INCPH cases. The major findings were observed in all of the INCPH cases in at least two (12%) portal tracts. The mean percentage of portal tracts with major findings was 58% (12–100%) in the INCPH group and was 15% (0–54%) in the control group (*p* < 0.001).

Minor findings such as portal vein herniation, hypervascularized portal tracts, and periportal abnormal vessels were seen in 13 INCPH cases (81%) and in 13 (72%) control group cases. The mean percentages of portal tracts with minor findings in INCPH and control groups were of 16% (0–38%) and 7% (0–18%), respectively (*p* = 0.014). Among INCPH cases, the mean percentage of portal tracts with minor findings was lower than the mean percentage of portal tracts with major findings (16% versus 58%, respectively, *p* < 0.005).

When major and minor findings were taken into consideration together, it was found that the percentage of portal tracts displaying major and/or minor diagnostic features was increased up to an average of 69% (19–100%) in INCPH cases and of 22% (8–54%) in control cases (*p* < 0.001).

Abnormal distribution of portal tracts, such as uneven proximity to each other was detected in 12 (75%) INCPH cases while none of the control group cases displayed this feature *(p* < 0.001). Sinusoidal dilatation observed in 6 cases (37.5%), in two of which were focal. Among the 16 INCPH cases, NRH was seen in 5 (31%) and none of the control cases showed NRH findings (*p* = 0.016).

In the evaluation of Masson trichrome stained slides, nine of the INCPH cases (56.2%) showed no fibrosis and seven cases (43.7%) displayed portal, pericellular, or pericentral fibrosis. Hypoplastic-rudimentary small portal tracts, which could be better visualized with MT staining, were observed in four (25%) cases.

**Fig. 1:**
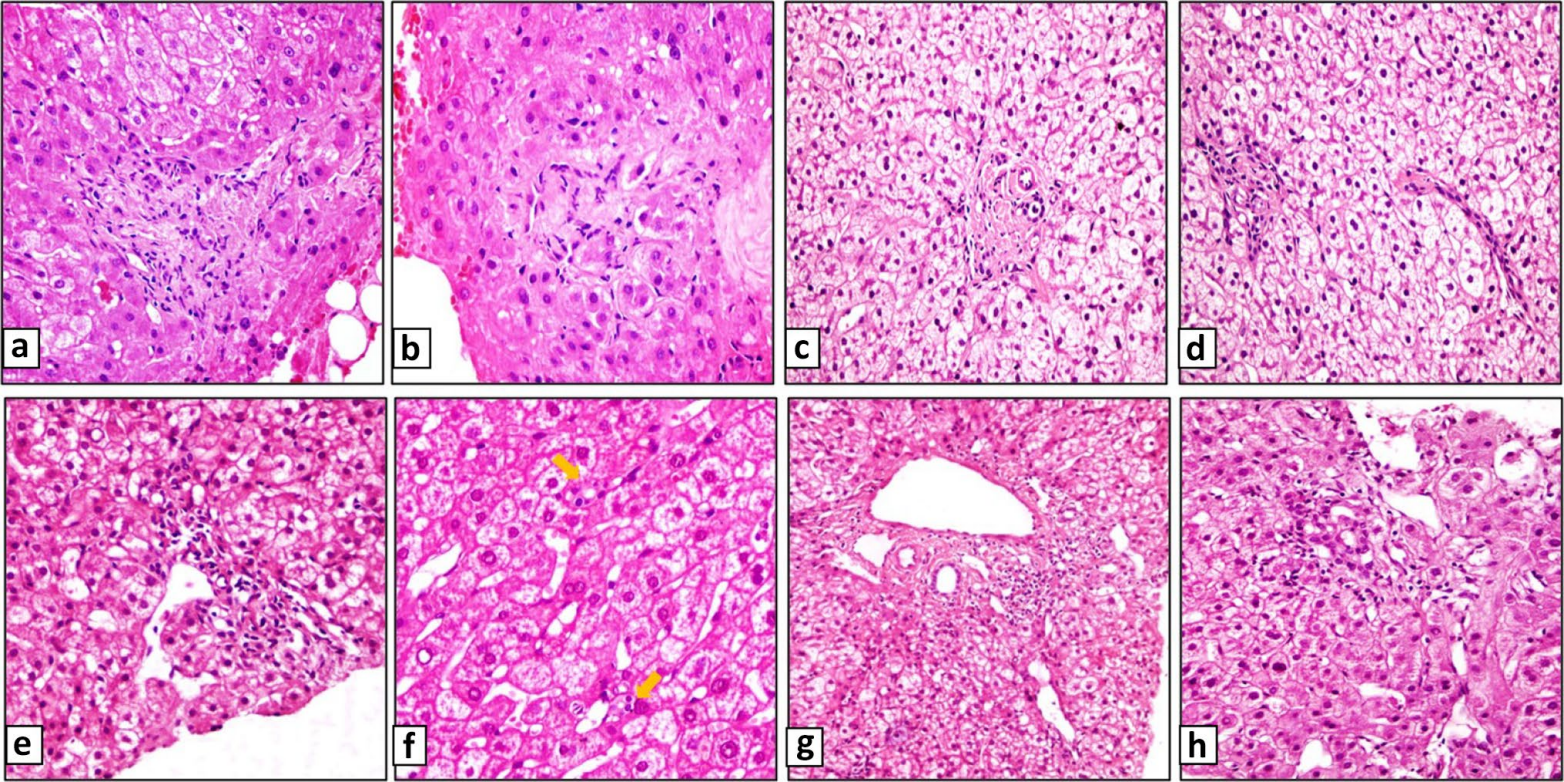
Histologic findings of INCPH. Portal vein obliteration, fibrotic portal tract (**a**, **b**), round shaped portal tract with portal vein loss (**c**), inconspicuous portal vein and close location of the portal tracts (**d**), portal vein herniation (**e**), hypoplastic small portal tracts marked with arrow (**f**), portal tract hypervascularization (**g**), and periportal abnormal vessels (**h**)

Major and minor histomorphologic findings are summarized in Table 2 and shown in Fig. 1.

**Table 2:** Histomorphologic findings, CD34 and glutamine synthetase immunohistochemical results in INCPH and control liver biopsies

INCPH cases	Number of PT	Number of PT with major findings^a^ *n* (%)	Number of PT with minor findings^b^ *n* (%)	Number of PT with major and minor findings *n* (%)	NRH	Fibrosis	Periportal CD34 stained area (µm)	GS
Case 1	9	4 (44)	1 (11)	5 (56)	Absent	Absent Small PTs	500	Normal
2	12	4 (33)	4 (33)	5 (43)	Absent	Absent	400	Normal
3	13	10 (77)	5 (38)	12 (92)	Absent	Absent Small PTs	400	Periportal, weak
4	12	11 (92)	1 (8)	11 (92)	**Present**	Dense portal fibrosis, round shaped PTs	500	Irregular
5	18	10 (56)	1 (6)	11 (61)	Absent	Absent Small PTs	400	Normal
6	40	18 (45)	6 (15)	24 (60)	Absent	Absent Small PTs	400	Normal
7	30	14 (47)	7 (23)	18 (60)	**Present**	Portal and pericellular fibrosis	600	Irregular, diffuse
8	17	17 (100)	0 (0)	17 (100)	Absent	Portal and pericentral fibrosis	500	Periportal, weak
9	12	12 (100)	0 (0)	12 (100)	Absent	Portal and pericellular fibrosis	400	Normal
10	7	5 (71)	1 (14)	6 (86)	Absent	Dense portal fibrosis, round shaped PTs	100	Periportal, weak
11	17	2 (12)	5 (29)	7 (41)	Absent	Rare portal fibrosis	200	Irregular
12	24	14 (58)	3 (16)	16 (67)	**Present**	Absent	300	Irregular, diffuse
13	15	9 (60)	4 (27)	12 (80)	Absent	Absent	400	Irregular
14	21	13 (62)	5 (24)	18 (86)	**Present**	Portal fibrosis	500	Irregular, diffuse
15	26	17 (65)	0 (0)	17 (65)	**Present**	Absent	100	Irregular
16	16	2 (13)	1 (6)	3 (19)	Absent	Absent	100	normal
Mean	18 (7–40)	58% (12–100%)	16% (0–38%)	69% (19–100%)			362	
Control cases								
Control 1	12	1 (8)	1 (8)	2 (17)	Absent	Absent	200	Normal
2	23	2 (9)	2 (9)	4 (17)	Absent	Absent	100	Normal
3	21	1 (5)	1 (5)	2 (10)	Absent	Absent	100	Normal
4	17	2 (12)	2 (12)	4 (24)	Absent	Absent	200	Normal
5	7	0 (0)	1 (14)	1 (14)	Absent	Absent	100	Normal
6	10	0 (0)	1 (10)	1 (10)	Absent	Absent	50	Normal
7	17	2 (12)	1 (6)	3 (18)	Absent	Absent	200	Normal
8	15	3 (20)	0 (0)	3 (20)	Absent	Absent	200	Normal
9	21	1 (5)	1 (5)	2 (10)	Absent	Absent	100	Normal
10	22	2 (9)	4 (18)	6 (27)	Absent	Absent	200	Normal
11	11	1 (9)	1 (9)	2 (18)	Absent	Absent	50	Normal
12	11	2 (18)	2 (18)	4 (36)	Absent	Absent	300	Normal
13	20	5 (25)	0 (0)	5 (25)	Absent	Absent	50	Normal
14	13	7 (54)	0 (0)	7 (54)	Absent	Absent	200	Normal
15	22	4 (18)	1 (5)	5 (23)	Absent	Absent	100	Normal
16	25	8 (32)	0 (0)	8 (32)	Absent	Absent	100	Normal
17	13	0 (0)	1 (8)	1 (8)	Absent	Absent	50	Normal
18	13	4 (31)	0 (0)	4 (31)	Absent	Absent	100	Normal
Mean	16 (7–25)	15% (0–54%)	7% (0–18%)	22% (8–54%)			133	

*NRH* nodular regenerative hyperplasia

^a^Major findings = PV narrowing/obliteration/or loss

^b^Minor findings = Herniation/hypervascularized portal tracts/periportal abnormal vessels

### Immunohistochemical analysis

*CD34* Periportal-periseptal sinusoidal CD34 expression was observed in 12 (75%) INCPH cases and only in one (5.6%) control case. The diameter of the periportal area with endothelial CD34 immunoexpression ranged between 100 and 600 µm (mean 362 µm, median 400 µm) and 50 to 300 µm (mean 133 µm, median 100 µm) in INCPH and control cases, respectively (p < 0.001).

*Glutamine synthetase* Irregular staining patterns such as periportal, enlarged pericentral, or multizonal staining were observed in 10 INCPH cases (62%), while the usual perivenular pattern was detected in all of the control cases (*p* < 0.001). Four of the 10 cases showed diffuse irregular staining covering zone 2 and zone 3 hepatocytes and these four cases were the ones exhibiting NRH features. Among cases representing NRH features, diffuse irregular staining was found in 80% (4 of 5 cases).

**Fig. 2:**
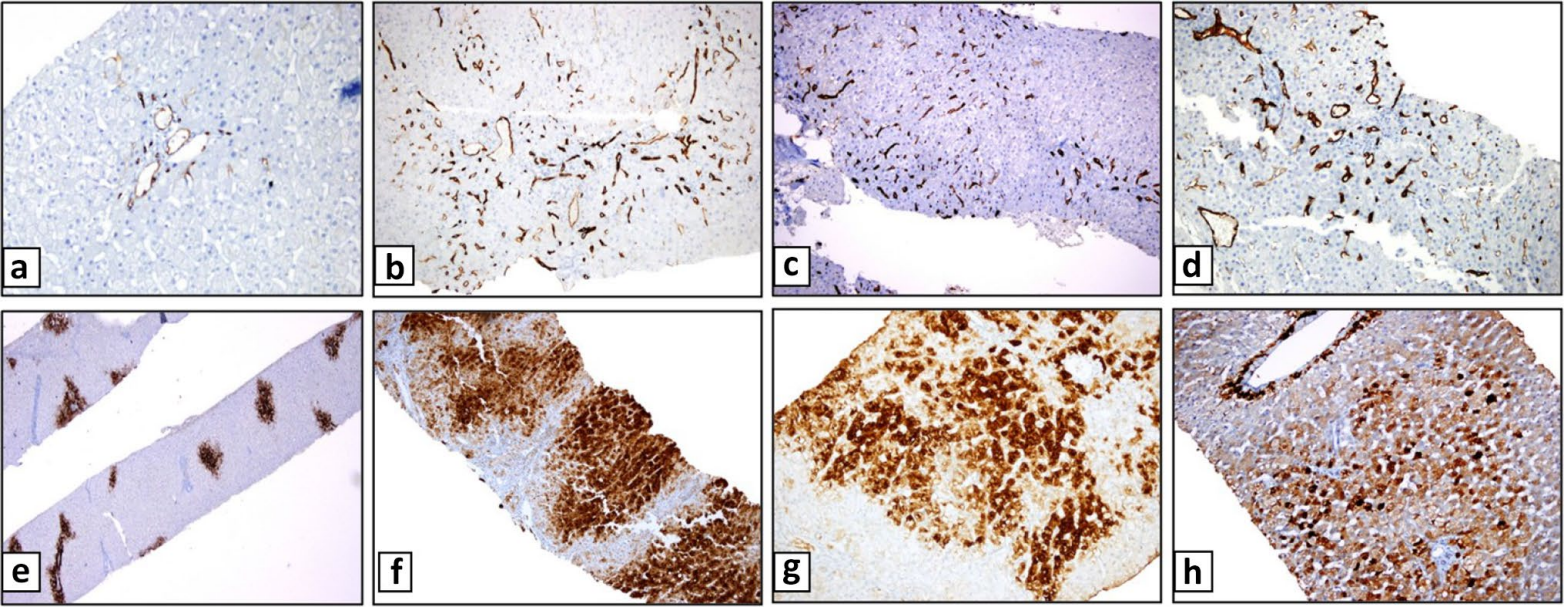
Staining of CD34 (**a**–**d**) and GS (**e**–**h**). CD34 immunoreaction in portal venule seen in control liver biopsy (**a**), periportal sinusoidal capillarization with CD34 in INCPH (**b**–**d**), normal GS staining in control liver biopsy (**e**), irregular (**f**–**h**), and multizonal (zone 2 and zone 3) GS staining in INCPH (**f**, **g**)

CD34 and glutamine synthetase immunohistochemistry findings are shown in Table 2 and Fig. 2**.**

The comparison of the histologic and immunohistochemical findings between INCPH and control groups is given in Table 3.

**Table 3:** The comparison of the histologic and immunohistochemical findings in INCPH and control group cases

	INCHP cases (mean ± STD)	Control group cases (mean ± STD)	*p*
Age (years)	34.69 ± 20.16	37.78 ± 10.84	0.576
Percentage of portal tracts with major findings (%)	58.43 ± 26.55	14.79 ± 13.82	< ***0.001***
Percentage of portal tracts with minor findings (%)	15.50 ± 12.33	7.01 ± 5097	***0.014***
Mean percentage of portal tracts with major and minor findings (%)	69.11 ± 23.28	21.80 ± 11.49	< ***0.001***
Widht of the periportal/periseptal area with CD34 expession (µm)	362.50 ± 158.64	133.33 ± 72.76	< ***0.001***

	INCHP cases *n*: 16	Control group cases *n*: 18	*p*
Proximity of portal tracts (%)	75.0	0	< ***0.001***
NRH (%)	31.3	0	***0.016***
Perisinusoidal capillarization (%)	75.0	5.6	< ***0.001***
Irregular GS staining (%)	62.5	0	< ***0.001***

Statistically significant *p*-values are designated in bold italics

## Discussion

Accurate histomorphologic diagnosis of INCPH is difficult because the histological findings are often ambiguous or heterogeneously distributed [32, 33]. We evaluated the histomorphologic findings in detail in liver biopsies of the patients clinically diagnosed as INCPH. Major findings (portal vein narrowing, obliteration, or loss) were observed in all cases in at least two portal tracts, while there were some cases in which minor findings (portal vein herniation, hypervascularized portal tracts, and periportal abnormal vessels) were absent. Furthermore, the number of portal tracts displaying major findings was higher than the number of those with minor findings. Portal venular changes which were formerly named as obliterative portal venopathy were the most common findings similar to the results of previous studies [3, 16, 18].

Histologic changes of INCPH can be subtle, patchy, and nonspecific, as they can be seen in a variety of other liver diseases [32, 34]. Certain and specific histologic criteria for diagnosis does not exist. Therefore, the definitive histopathologic diagnosis of INCPH were challenging and need to be focused on. In the study of Liang et al., a cut-off point of 8.3% for portal tracts exhibiting portal vein sclerosis was shown as optimal for the diagnosis of INCPH [33]. Even though the mean percentage of portal tracts displaying major findings was 58%, it was as low as 12%, in our study cohort. Another study investigating the prevalence of histological features of INCPH in general population revealed that at least one feature of INCPH was noted in 90% of the study cases [34]. Similarly, in our study, all of the control biopsies showed at least one histologic feature. However, when major and minor findings were taken into consideration together, the amount of portal tracts displaying these findings was lower than the INCPH cases (22% vs. 69%, respectively). These results may indicate that the frequency of findings might be more important than their presence in only a few portal tracts and a certain cut-off point of the percentage of portal tracts with major and minor findings might be useful for decision making in pathologic diagnosis. We might consider a cut of point of 12% for major findings, depending on our results. However, further studies including larger series need to be performed for such a conclusion.

Portal tract remnants and NRH were found to be significant positive predictors for the diagnosis, such as portal venopathy [35]. We detected portal tract remnants and NRH features in some of our cases. NRH was regarded as a subtle finding and diagnosis of NRH may be challenging on needle biopsies [33]. Guilbert et al. showed abnormal zone 2 immunoreaction with GS in majority of their NRH cases [29]. We observed irregular GS staining in most cases and four of them exhibiting NRH features had diffuse irregular staining. Irregular GS staining seemed to be a helpful diagnostic finding, especially in cases with NRH.

Considering the possibility of using an immunohistochemical marker to provide more reliable and less variable findings for pathologic diagnosis of INCPH in biopsy samples, we examined the CD34 immunohistochemistry and observed increased periportal-periseptal endothelial expression and found that the periportal-periseptal area showing endothelial CD34 immunoexpression was wider in the INCPH cases than in the control cases. As a similar finding, Bakshi et al. showed CD34 positivity in periportal sinusoidal endothelial cells extending to midzonal areas in 22 histologically diagnosed NRH cases [25]. Increased immunoreactivity for CD34 was found in sinusoidal endothelial cells radiating from portal tract-like structures in focal nodular hyperplasia and macroregenerative nodules because of the altered circulation [23]. Our results suggest that periportal sinusoidal capillarization shown by CD34 immunohistochemistry may be a helpful diagnostic feature for INCPH.

## Conclusion

Portal vein narrowing, obliteration, or loss, herniated portal veins, hypervascularized portal tracts, and periportal abnormal vessels are commonly observed histologic findings in INCPH although they can be seen in the liver biopsies of healthy individuals. It is also remarkable that the increased number of portal tracts displaying histologic findings may be useful and reliable for diagnosis. We conclude that the detection of periportal sinusoidal capillarization by CD34 immunohistochemistry, along with the histologic findings may increase diagnostic accuracy. Irregular GS staining especially in the presence of NRH, might also be a supportive finding.

## Data Availability

The authors declare that the data can be available upon the request of the journal.

## References

[1] Bloom S, Kemp W, Lubel J (2015). Portal hypertension: pathophysiology, diagnosis and management. Intern Med J.

[2] Garcia-Tsao G (2005). Portal hypertension. Curr Opin Gastroenterol.

[3] Kmeid M, Liu X, Ballentine S, Lee H (2021). Idiopathic non-cirrhotic portal hypertension and porto-sinusoidal vascular disease: review of current data. Gastroenterology Res.

[4] Mikkelsen WP, Edmondson HA, Peters RL, Redeker AG, Reynolds TB (1965). Extra- and intrahepatic portal hypertension without cirrhosis (hepatoportal sclerosis). Ann Surg.

[5] European Association for the Study of the Liver (2016). EASL clinical practice guidelines: vascular diseases of the liver. J Hepatol.

[6] Hillaire S, Bonte E, Denninger MH, Casadevall N, Cadranel JF, Lebrec D (2002). Idiopathic non-cirrhotic intrahepatic portal hypertension in the West: a re-evaluation in 28 patients. Gut.

[7] Schouten JN, Garcia-Pagan JC, Valla DC, Janssen HL (2011). Idiopathic noncirrhotic portal hypertension. Hepatology.

[8] Cazals-Hatem D, Hillaire S, Rudler M, Plessier A, Paradis V, Condat B (2011). Obliterative portal venopathy: portal hypertension is not always present at diagnosis. J Hepatol.

[9] Okudaira M, Ohbu M, Okuda K (2002). Idiopathic portal hypertension and its pathology. Semin Liver Dis.

[10] Sarin SK, Kapoor D (2002). Non-cirrhotic portal fibrosis: current concepts and management. J Gastroenterol Hepatol.

[11] Nakanuma Y, Hoso M, Sasaki M, Terada T, Katayanagi K, Nonomura A (1996). Histopathology of the liver in non-cirrhotic portal hypertension of unknown aetiology. Histopathology.

[12] Guido M, Sarcognato S, Sonzogni A, Luca MG, Senzolo M, Fagiuoli S (2016). Obliterative portal venopathy without portal hypertension: an underestimated condition. Liver Int.

[13] Guo T, Qian J, Zhu L, Zhou W, Zhu F, Sun G, Fang X (2012). Clinical analysis of 15 cases of liver nodular regenerative hyperplasia. Cell Biochem Biophys.

[14] Al-Mukhaizeem KA, Rosenberg A, Sherker AH (2004). Nodular regenerative hyperplasia of the liver: an under-recognized cause of portal hypertension in hematological disorders. Am J Hematol.

[15] Naber AH, Van Haelst U, Yap SH (1991). Nodular regenerative hyperplasia of the liver: an important cause of portal hypertension in non-cirrhotic patients. J Hepatol.

[16] Lee H, Rehman AU, Fiel MI (2016). Idiopathic noncirrhotic portal hypertension: an appraisal. J Pathol Transl Med.

[17] Guido M, Sarcognato S, Sacchi D, Colloredo G (2018). Pathology of idiopathic non-cirrhotic portal hypertension. Virchows Arch.

[18] Fiel MI, Schiano TD (2019). Idiopathic noncirrhotic portal hypertension. Semin Diagn Pathol.

[19] Guido M, Alves VAF, Balabaud C, Bhathal PS, Bioulac-Sage P, Colombari R (2019). Histology of portal vascular changes associated with idiopathic non-cirrhotic portal hypertension: nomenclature and definition. Histopathology.

[20] Aggarwal S, Fiel MI, Schiano TD (2013). Obliterative portal venopathy: a clinical and histopathological review. Dig Dis Sci.

[21] Wanless IR (2020). The role of vascular injury and congestion in the pathogenesis of cirrhosis: the congestive escalator and the parenchymal extinction sequence. Curr Hepatol Rep.

[22] Wanless IR (1990). Micronodular transformation (nodular regenerative hyperplasia) of the liver: a report of 64 cases among 2,500 autopsies and a new classification of benign hepatocellular nodules. Hepatology.

[23] Theuerkauf I, Zhou H, Fischer HP (2001). Immunohistochemical patterns of human liver sinusoids under different conditions of pathologic perfusion. Virchows Arch.

[24] Zhang X, Thomas C, Schiano TD, Thung SN, Ward SC, Fiel MI (2020). Aberrant von Willebrand factor expression of sinusoidal endothelial cells and quiescence of hepatic stellate cells in nodular regenerative hyperplasia and obliterative portal venopathy. Histopathology.

[25] Bakshi N, Gulati N, Rastogi A, Chougule A, Bihari C, Jindal A (2020). Nodular regenerative hyperplasia—an under-recognized vascular disorder of liver. Pathol Res Pract.

[26] Bioulac-Sage P, Rebouissou S, Thomas C, Blanc JF, Saric J, Sa Cunha A (2007). Hepatocellular adenoma subtype classification using molecular markers and immunohistochemistry. Hepatology.

[27] Di Tommaso L, Franchi G, Park YN, Fiamengo B, Destro A, Morenghi E (2007). Diagnostic value of HSP70, glypican 3, and glutamine synthetase in hepatocellular nodules in cirrhosis. Hepatology.

[28] Bioulac-Sage P, Laumonier H, Rullier A, Cubel G, Laurent C, Zucman-Rossi J, Balabaud C (2009). Over-expression of glutamine synthetase in focal nodular hyperplasia: a novel easy diagnostic tool in surgical pathology. Liver Int.

[29] Guilbert MC, Therrien A, Soucy G, Trudel D, Nguyen BN (2020). Nodular regenerative hyperplasia: expression pattern of glutamine synthetase and a potential role for hepatic progenitor cells. Appl Immunohistochem Mol Morphol.

[30] Gebhardt R, Baldysiak-Figiel A, Krugel V, Ueberham E, Gaunitz F (2007). Hepatocellular expression of glutamine synthetase: an indicator of morphogen actions as master regulators of zonation in adult liver. Prog Histochem Cytochem.

[31] Sato Y, Harada K, Sasaki M, Nakanuma Y (2015). Altered intrahepatic microcirculation of idiopathic portal hypertension in relation to glutamine synthetase expression. Hepatol Res.

[32] Kmeid M, Zuo C, Lagana SM, Choi WT, Lin J, Yang Z (2020). Interobserver study on histologic features of idiopathic non-cirrhotic portal hypertension. Diagn Pathol.

[33] Liang J, Shi C, Dupont WD, Salaria SN, Huh WJ, Correa H (2021). Key histopathologic features in idiopathic noncirrhotic portal hypertension: an interobserver agreement study and proposal for diagnostic criteria. Mod Pathol.

[34] Zuo C, Chumbalkar V, Ells PF, Bonville DJ, Lee H (2017). Prevalence of histological features of idiopathic noncirrhotic portal hypertension in general population: a retrospective study of incidental liver biopsies. Hepatol Int.

[35] Verheij J, Schouten JN, Komuta M, Nevens F, Hansen BE, Janssen HL, Roskams T (2013). Histological features in western patients with idiopathic non-cirrhotic portal hypertension. Histopathology.

